# A Comprehensive Review of Radiotherapy-Induced Coronary Artery Disease—Epidemiology, Biological Mechanisms, and Preventive Strategies

**DOI:** 10.3390/ijms26115401

**Published:** 2025-06-04

**Authors:** Jalil Daher, Antonio Rizza, Alessandro Tonacci, Andrea Borghini

**Affiliations:** 1 Department of Biology, Faculty of Arts and Sciences, University of Balamand, Balamand 100, Lebanon; jalil.daher@balamand.edu.lb; 2 U.O.C. Cardiologia Diagnostica e Interventistica, Fondazione Toscana Gabriele Monasterio, 54100 Massa, Italy; antonio.rizza@ftgm.it; 3 CNR Institute of Clinical Physiology, 56124 Pisa, Italy; alessandro.tonacci@cnr.it

**Keywords:** radiotherapy, coronary artery disease, radiation-induced coronary artery disease, cellular and molecular mechanisms

## Abstract

Radiation-induced cardiac toxicity is a recognized complication in patients undergoing thoracic radiotherapy. A crucial manifestation of this toxicity is the damage caused to coronary arteries, which can result in accelerated atherosclerosis that may remain undetected for many years. As cancer survival rates continue to improve, the incidence of radiation-induced coronary artery disease (RICAD) is increasing, making it one of the leading causes of morbidity and mortality among patients treated with radiotherapy for mediastinal cancers. The pathophysiology of RICAD involves a complex interplay of cellular mechanisms, including endothelial dysfunction, inflammation, and fibrosis. These processes are related to several molecular insults such as DNA damage, telomere erosion, and mitochondrial dysfunction. However, to fully understand the initiation and progression of the disease, further research is critical to uncover additional contributing factors. Different strategies for preventing cardiovascular complications in cancer patients are gaining significant attention. Recent advancements in radiotherapy, particularly the new FLASH radiotherapy technique, show promise in reducing the incidence of these complications. This review focuses on the effects of radiotherapy on coronary artery disease, exploring the underlying cellular and molecular mechanisms, as well as potential strategies to prevent RICAD.

## 1. Introduction

The incidence and mortality rates of cancer are rising as life expectancies improve, with 19.3 million new cancer cases diagnosed worldwide in 2020 [1]. Approximately 50% of oncology patients require radiotherapy at some point during their treatment, resulting in a growing population of post-radiotherapy patients [2]. Radiotherapy can be administered alone or in conjunction with surgery and systemic treatments for a variety of cancers, aiming to optimize tumor control and enhance quality of life while minimizing toxicity and preserving surrounding organs. The side effects of radiotherapy vary depending on the anatomical area being treated and are influenced by factors such as the total radiation dose, dose per fraction, proximity to sensitive tissues and organs, and interactions with other cancer treatments [3].

High-dose radiation exposure to the thorax and heart is primarily used in the context of adjuvant radiation therapy following breast surgery, as well as in the treatment of lung and oesophageal cancers, and as an adjunct to systemic therapy for lymphoma. While radiation therapy has significantly enhanced the survival rates and longevity of patients with thoracic malignancies [4,5], the collateral damage to surrounding tissues has resulted in unavoidable complications that can undermine the overall effectiveness of treatment.

Radiation-induced heart disease (RIHD) is increasingly recognized as a major cause of morbidity and mortality among cancer survivors. High doses of radiation can result in premature cardiovascular disease (CVD) in these individuals, often occurring years after their treatment [6,7]. The risk of radiation-induced complications is further increased by the combined effects of chemotherapy and cardiovascular risk factors, such as diabetes, hypertension, obesity, and dyslipidaemia. The estimated incidence of RIHD is reported to be between 10% and 30% within 5 to 10 years post-treatment, with variability depending on the timing of radiotherapy and the type of cancer involved [8].

Radiation-induced cardiac dysfunction covers coronary artery disease (CAD), pericarditis, cardiomyopathy, valvular heart disease (VHD), and cardiac conduction abnormalities [6,9]. CAD represents the most common manifestation of radiation-induced cardiovascular disease, with an incidence of up to 85% [10]. Accelerated CAD resulting from ionizing radiation exposure is referred to as “radiation-induced coronary artery disease” (RICAD). Long-term studies involving survivors of nuclear explosions and nuclear workers [11,12], and patients who have undergone radiotherapy provide valuable insight into RICAD, with the latter group being the primary contributor to its incidence [13].

As cancer survival rates improve, the incidence of RICAD is on the rise, making it the second most prevalent cause of morbidity and mortality among patients who have received radiotherapy for mediastinal cancers. RICAD often presents atypically or may even be asymptomatic, typically manifesting after a latency period of approximately 15 years post-radiotherapy. Efficacious strategies for predicting and preventing cardiovascular toxicities in cancer patients are gaining increasing attention, as highlighted by the most recent ESC guidelines [14]. In this review, we describe the effects of radiotherapy on CAD and the cellular and molecular mechanisms involved, along with potential preventive approaches to minimize RICAD.

## 2. Radiotherapy-Induced Coronary Artery Disease: Epidemiology

As already mentioned, radiation-induced cardiac dysfunction, or what is also known as RIHD, encompasses a wide spectrum of clinical manifestations affecting the heart. However, the overall burden of this disease has been relatively underestimated due to discrepancies in the differential survival rates of thoracic cancer patients undergoing radiotherapy [15]. Accordingly, most research on the prevalence of RIHD centers on cancer patients with the highest 5-year survival rates, which include patients with Hodgkin’s lymphoma and breast cancer. On this note, it is worth mentioning that the incidence rates and data extracted from such research vary greatly depending on the assessment tools that were applied in relevant studies which include mainly the duration of the follow-up time in patients. For instance, the frequency of heart complications in breast cancer survivors varies a lot among different studies and when compared to epidemiological data from lymphoma patients, i.e., 0.5% to 37% vs. 49.5 to 54.6%, respectively, with the higher incidence rates seen in Hodgkin’s lymphoma cancer patients [15,16,17]. Overall, CVD is considered to be the leading cause of mortality in Hodgkin’s lymphoma and breast cancer survivors who were treated with radiotherapy, accounting for up to 56% of non-cancer deaths in those groups of patients [18]. The most common manifestation of radiation-induced CVD is CAD, with an incidence rate reaching 85% [10]. For instance, it has been shown that the 10-year cumulative incidence of CAD hospitalizations in middle-aged women treated for breast cancer is significantly increased [19]. It has also been shown that cancer patients who underwent thoracic radiation for the treatment of Hodgkin’s lymphoma were at 2.5-fold increased risk of developing fatal myocardial infarction when compared to the general population [20].

Interestingly, autopsy studies have revealed that the latter complications even occurred in very young 15 years old subjects undergoing mediastinal radiation. Also, other studies including lymphoma patients have shown that the incidence of CAD is significantly increased to around 59% in lymphoma survivors [21]. In the United States, cancer survivors were reported to exhibit an increase in cardiovascular mortality during the last couple of decades, with up to a 3.6-fold increase in developing CAD in comparison with the general population [22,23]. Lately, there has been a significant rise in the survival of young adults with cancer, more specifically, of those with Hodgkin’s lymphoma, which is among the most common types of cancer in this age group. In this population of patients, the estimated CAD cumulative incidence is around 60% forty years after radiotherapy; in addition, young lymphoma survivors were at a higher risk of developing cardiac complications such as myocardial infarction when matched with their unaffected siblings.

Ultimately, mediastinal radiation beyond 42 Gy increased cardiac-related deaths in this group of patients by around 41-fold [23,24,25]. In one radiotherapy-related epidemiological study concerning Hodgkin’s lymphoma, the cumulative incidence rates for various cardiovascular complications in radiotherapy-treated patients were all significantly increased; after a period of 40 years follow-up, patients that were subjected to radiotherapy before the age of 25 had cumulative incidence rates of 11%, 20%, and 31% for heart failure (HF), CAD, and VHD, respectively [17]. In another meta-analysis study, cardiac mortality was significantly increased in breast cancer patients undergoing radiotherapy [26]. In parallel, Hodgkin’s lymphoma patients receiving radiotherapy exhibited a 7.4% increase in the risk of coronary heart disease per 1 Gy of radiation [25].

## 3. Radiotherapy-Induced Coronary Artery Disease: The Underlying Biological Mechanisms

### 3.1. Focus on Molecular Mechanisms: Nuclear and Mitochondrial Damage

Radiation-induced cardiotoxicity is related to a variety of molecular and cellular insults. Ionizing radiation causes DNA damage both directly and indirectly, primarily through reactions with reactive oxygen species (ROS). ROS randomly interact with macromolecules, such as DNA, proteins, and lipids, across all cellular compartments, including the nucleus and mitochondria. The nature of these interactions, the radiation energy (whether low-LET or high-LET), and the absorbed dose of ionizing radiation influence the severity and extent of damage to cellular components. This, in turn, leads to various cellular events, including senescence and apoptosis, each associated with significant pathological implications [27]. At the DNA level, this damage can manifest as base mismatches, base modifications, single-stranded breaks (SSBs), double-stranded breaks (DSBs), and cross-links. These types of lesions can arise independently or concurrently, leading to complex patterns of DNA damage [28].

The occurrence of DSBs, recognized as the most deleterious form of DNA damage, activates various cellular factors, resulting in the phosphorylation of the histone variant H2AX, which leads to the formation of γH2AX. To explore the connection between radiation-induced atherosclerosis and the senescence-associated secretory phenotype (SASP) in carotid arteries, apolipoprotein E knockout mice were subjected to irradiation with 6 Gy of γ-rays. This treatment resulted in elevated levels of DNA damage markers and senescence-associated proteins, thereby accelerating plaque formation and atherogenesis [29]. Additionally, in vitro experiments demonstrated that exposure to 4 Gy of γ-rays activated key signaling pathways in DNA damage response, including ATM, CHK2, and p53/p21, along with an increase in senescence-associated beta-galactosidase activity in human umbilical vein endothelial cells (HUVECs) [30]. Furthermore, 10 Gy of γ-rays induced a significant inflammatory response and upregulated the expression of the protein DDB2 in human cardiac endothelial cells [31].

**Figure 1:**
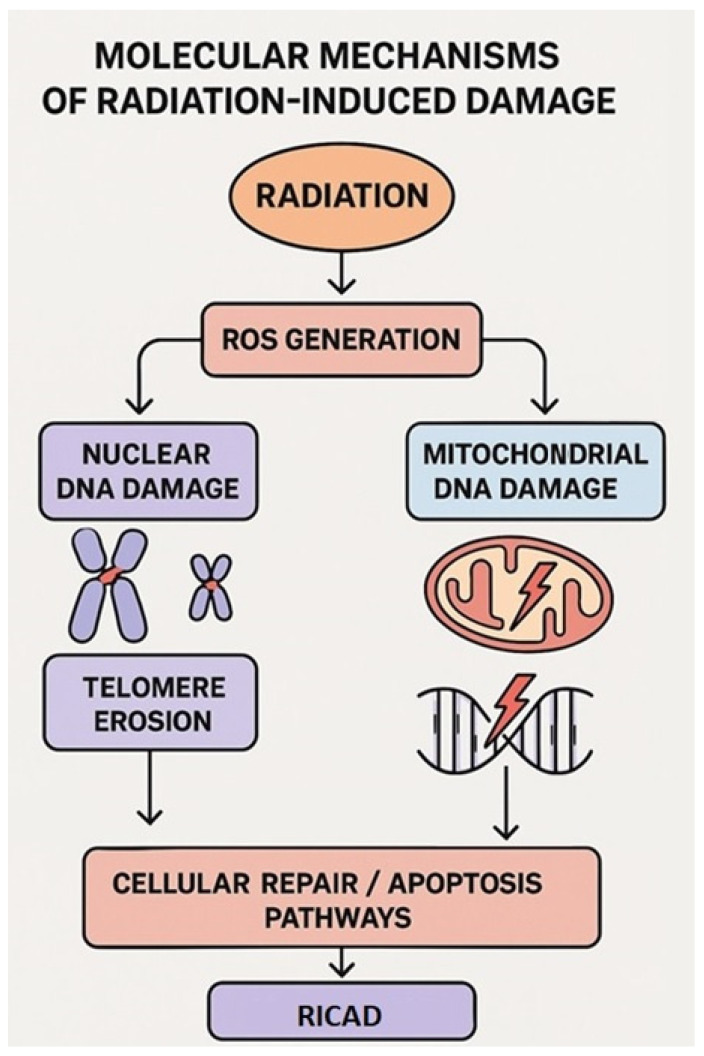
Molecular mechanisms of radiation-induced damage. Infographic showing ROS generation, nuclear vs. mitochondrial DNA damage, telomere erosion, and the activation of cellular repair/apoptosis pathways, as well as their role in radiation-induced coronary artery disease.

Radiation-induced ROS generation can result in telomere shortening and destabilization of the mitochondria [32,33,34]. Telomere shortening contributes to cellular senescence, which contributes to atherogenesis and CVD [35,36] (Figure 1). This leads to the question of whether radiation-associated cardiac pathological modifications may be partially attributed to telomere shortening.

The exposure to high-dose γ-ray irradiation has been shown to reduce telomere length, telomerase activity, the expression of telomerase reverse transcriptase, and the levels of telomere-binding proteins such as TPP1 and POT1 in human fibroblasts [37]. In a study conducted by Berardinelli et al., telomere alterations were observed in human fibroblasts subjected to both 4 Gy of low-linear energy transfer (LET) radiation (X-rays) and high-LET radiation (28.5 keV/μm protons) at various time intervals post-irradiation [38].

Furthermore, the telomere/telomerase system appears to safeguard chromosomal integrity in X-ray-irradiated human fibroblasts by facilitating repair processes during the G2 phase of the cell cycle [39]. In the context of in vivo studies, Maeda et al. found no significant impact of radiotherapy on telomere length in peripheral leukocytes from cancer patients [40]. On the contrary, M’Kacher et al. [41] demonstrated for the first time significant telomere shortening in a large cohort of patients treated with radiotherapy who subsequently developed CVD. Notably, telomere length appears to be an independent prognostic factor that may help identify patients at high risk for developing CAD [41].

Mitochondria are the primary source of cellular energy and are present in various cell types. Their role is particularly crucial in the heart, which has high energy demands that are met through oxidative phosphorylation. Dysfunction in mitochondria is linked to the development of several cardiac diseases, including atherosclerosis, primarily due to the excessive production of ROS [42]. Mitochondrial DNA (mtDNA) is a significant target for radiation damage due to its lack of protective histones. Furthermore, mtDNA is typically repaired less efficiently than nuclear DNA and exhibits a mutation rate that is 10 to 1000 times higher than that of nuclear DNA, making it an excellent model for investigating the mutational effects of radiation [43]. mtDNA alterations are involved in the processes that initiate or exacerbate mitochondrial dysfunction, influencing biological functions and affecting human health [44].

Research studies have highlighted the role of radiation-induced mitochondrial dysfunction and its subsequent effects on various cardiac functions, both in preclinical models and in human studies [45]. In the late 1960s, it was found that radiation significantly affects mitochondrial structure, both in the short and in the long term. For instance, seven years after a human patient received high-dose radiation (52 Gy) to the mediastinal area, electron microscopy revealed swollen cardiomyocyte mitochondria with fewer cristae and fused outer membranes [46].

Barjaktarovic et al. [47] conducted a proteomic analysis of cardiac mitochondria from thoracic-irradiated C57BL/6 mice at 4 weeks post-exposure to varying doses. The higher dose of 2 Gy resulted in the downregulation of different proteins associated with oxidative phosphorylation, pyruvate metabolism, and cytoskeletal structures [47]. Mice exposed to this dose demonstrated functional impairments, including decreased succinate-driven respiratory capacity, increased ROS levels and protein oxidation in mitochondria. Notably, reduced mitochondrial respiratory capacity was observed at 40 weeks post-exposure, indicating ongoing oxidative stress-related damage to mitochondria [48]. Research in mice has also demonstrated radiation-induced mitochondrial changes related to oxidative stress and respiratory capacity lasting from hours to months after exposure. Briefly, mice exposed to 3 Gy of total body irradiation exhibited immediate cardiac structural and functional changes, with mitochondrial proteins most sensitive to ionizing radiation [49].

Baselet et al. demonstrated that dysregulation of the Bcl2 pathway leads to endothelial inflammation, apoptosis, and senescence, contributing to atherosclerosis [50]. Both in vitro and in vivo studies have shown evidence of endothelial cell senescence after radiation exposure. HUVECs exhibited altered mitochondrial membrane potential two days after irradiation with doses of 1.5, 4, and 10 Gy. While the membrane potential returned to baseline levels by days 5 and 6 for the 1.5 and 4 Gy doses, cells exposed to the higher dose maintained a reduced mitochondrial activity [50].

Overall, while ionizing radiation induces telomere erosion and mitochondrial dysfunction, the variations in response to radiation, such as LET, different doses, and dose rates, remain to be fully investigated in relation to RICAD.

### 3.2. Focus on Cellular Mechanisms

Emerging studies have clearly shown that the cellular responses to irradiation are complex and that the major elements which are responsible for the development of RICAD remain to be elucidated. Beyond the mechanisms summarized above, it is becoming more evident nowadays that irradiation-related oxidative stress could be also responsible for the activation of various cellular processes that are linked to inflammation and endothelial cell activation. For instance, an increase in ROS generation and mitochondrial stress within the cell can trigger both the innate and adaptive immune response, as well as many secondary effects, including apoptosis, cellular senescence, and fibrosis [32,51]. Meanwhile, many products that are generated by affected cells in response to radiation can behave as danger-associated molecular patterns (DAMPs), inducing cardiotoxicity by relying on both inflammatory pathways and other cellular mechanisms that involve senescence [52].

Of note, it was shown that cardiomyocytes are more resistant to the effects of radiation when compared to endothelial cells in the coronary arteries of the heart tissue. Thus, it is thought that radiation-mediated cardiotoxicity and cardiac injury by itself may be closely related to the endothelial cell dysfunctional phenotype that is induced as a consequence of radiotherapy, and which is connected in many intricate ways to the initiation and progression of atherosclerosis and all its subsequent complications [53,54]. Depending on the dose and mode of radiation and the specific location of endothelial cells within the subjected organ, these cells can respond to irradiation in many different ways. Particularly, endothelial cells that reside in coronary arteries undergo senescence on the long-term after irradiation. They also exhibit a high rate of non-apoptotic cell death when exposed to fractionated radiation therapy. Conversely, it was shown that those same cells experience apoptosis when subjected to increasing fractional radiation doses [55].

Notably, endothelial cells are not the only types of cells that play a role in this particular context; endothelial progenitor cells are also present in the arterial wall of blood vessels, where their functions are not exclusive to vascular remodeling but also relate to vascular dysfunction under pathological conditions. Interestingly, it has been shown that increasing doses of radiation have differential effects on those two types of cells; while higher doses induce senescence and a premature atherosclerotic phenotype in endothelial cells, the same levels lead to cell cycle arrest (mediated by p21) and apoptosis (mediated by Bax) in endothelial progenitor cells [56]. Of note, senescent endothelial cells play a pivotal role in the manifestation of endothelial dysfunction seen during the initiation of atherosclerosis. Senescence may be due either to aging and telomere shortening—called replicative senescence—or to DNA damage and oncogenic triggers. Also, senescence caused by irradiation and involving DNA damage shares different mechanisms compared to replicative or oncogene-stimulated senescence [57]. Meanwhile, the mechanism of senescence in endothelial cells is linked to their SASP, which is, in turn, associated with the production of multiple modulators, including cytokines, that are involved in the chronic inflammatory state observed under relevant pathological conditions. In fact, radiation doses ranging from 2 to 4 Gy are responsible for increasing senescence-associated markers, mainly β-galactosidase, in HUVECs. On the other hand, higher doses, reaching up to 10 Gy, can lead to apoptosis in these cells, as reflected by increased annexin V staining. Conversely, the same doses can trigger senescence in adult endothelial cells, suggesting that the mode of death in endothelial cells and their response to irradiation may depend on the degree of maturation of these cells, as well as the radiation dose they receive [56,58].

Radiation-induced senescence in endothelial cells might be partly linked to the inactivation of the Akt/PI3K/mTOR pathway, as well as the upregulation of CD44 expression on senescent endothelial cells. The latter marker is an adhesion protein that is involved in promoting the adhesion of monocytes to endothelial cells, a key step during atherogenesis. The effects of radiation at this level are not limited to CD44 expression only; they encompass a wide range of other endothelial cell receptors, including integrins, selectins, and immunoglobulin superfamily receptors such as vascular cell adhesion molecule-1 (VCAM-1) and intercellular adhesion molecule-1 (ICAM-1), which are all known to be involved in chronic inflammatory reactions and the development of atherosclerosis [54]. On a related note, a major factor that has been shown to be upregulated in HUVECs after exposure to radiation is nuclear factor-kappa beta (NF-κB), which is considered to be the most crucial player in orchestrating a wide variety of inflammatory reactions in the cell. It is thought that the increased accumulation of senescent cells might overwhelm tissues and could lead to a plethora of inflammatory products, including chemokines, pro-inflammatory cytokines, and extracellular proteases, all part of the SASP and excessively upregulated during radiation-induced vascular dysfunction [59]. For instance, HUVECs exposed to radiation show a significant increase in the levels of pro-inflammatory cytokines such as IL-6 and IL-8. Furthermore, in rats, whole-body irradiation was associated with an upregulation of major inflammatory cytokines, including IL-6, IL-1, and TNF-α [54]. Notably, all these cytokines are instrumental during the development of atherosclerosis as they are tightly linked to various pro-atherogenic mechanisms that involve the recruitment of immune cells, mainly macrophages, to the site of inflammation and the production of ROS, culminating in a heightened state of oxidative stress. In fact, studies have shown that radiation can trigger ROS-induced endothelial dysfunction and injury that mimic what happens during myocardial ischemia [60]. Irradiation also contributes to impairment in NO-mediated vasomotor functions and stenosis in human cervical arteries. Finally, radiation-induced ROS production was shown to be associated with the generation of peroxynitrite species that are responsible for the nitrosylation of tyrosine residues in proteins and other lipoprotein particles such as LDL, impairing their functions.

Additionally, oxidative stress can lead to the lipid peroxidation of phospholipids in the plasma membrane of cardiomyocytes, hindering their contractile capacity and enhancing the dysfunctional phenotype [54,61]. Interestingly, oxidative stress and inflammatory mechanisms activated by radiation are implicated in blood hemostasis, where they activate platelets through the downregulation of thrombomodulin, creating a pro-thrombotic environment. Similarly, it has been reported that radiation can upregulate the expression of the von Willebrand factor in endothelial cells, contributing to a further increase in blood clotting and a hypercoagulable environment after irradiation. Additionally, one of the major anti-fibrinolytic factors, plasminogen activator inhibitor-1 (PAI-1), was shown to be highly involved in radiation-induced endothelial dysfunction and the ensuing prothrombotic state; in fact, abrogating the function of PAI-1 in endothelial cells has been proven to significantly reduce radiation-induced cell death [62,63]. Alongside the processes discussed above, emerging insights into the mechanisms behind RICAD have highlighted tissue fibrosis as a potential process responsible for the cardiovascular effects induced by radiotherapy. Actually, endothelial cell injury and activation lead to the secretion of multiple interleukins and chemotactic factors, as well as pro-fibrotic cytokines, including PDGF, TGF-β, CTGF, and bFGF [64,65]. Concurrently with the effects of other inflammatory mediators, the latter factors are responsible for the onset of radiation-induced fibrotic mechanisms that encompass the irregular production and deposition of collagen and collagenase enzymes, as well as other proteases within the extracellular matrix of affected tissues. Furthermore, mediators of inflammation, including TGF-β, IL-4, and IL-13, play a crucial role in the activation of fibroblasts and the changes in the extracellular matrix composition seen during fibrosis. Thus, chronic inflammation is being reassessed for its predominant role as a mediator of fibrosis in this particular context [66].

Overall, although various mechanisms have been tackled to better understand RICAD etiology, the latter pathology still warrants further research that could be essential in delineating some potential additional processes and denominators that may play a role in the initiation and progression of the disease.

## 4. Shielding the Heart: Potential Preventive Strategies in Radiotherapy-Induced Coronary Artery Disease

Recent advancements in radiotherapy techniques can significantly minimize the cardiac dose during treatment. Given that the primary factor in radiation-induced cardiac damage is the incidental radiation exposure to heart tissue in thoracic cancer patients undergoing radiotherapy, these innovations focus on reducing heart irradiation. This is achieved through meticulous planning of the treatment schedule and delivery methods for radiation therapy. Thus, traditional radiation methods were first replaced with three-dimensional conformal radiation therapy (3DCRT), which relies on computed tomography technology for more accurate delivery and thus fewer off-target effects [67]. In addition to the latter method, an even more advanced technique has been developed due to further technological advances in the field; this method is actually called intensity-modulated radiation therapy (IMRT), which can simultaneously deliver multiple radiation beams with variable individual intensities that are very efficient in targeting irregularly shaped tumor masses and, consequently, sparing non-target tissues. Notably, volumetric modulated arc therapy (VMAT) is a variant of IMRT that was introduced a couple of decades ago to further improve the efficacy of IMRT. Additionally, the deep-inspiratory breath hold (DIBH) technique is now routinely employed for the treatment of left-sided breast cancer. This method effectively displaces the breast and chest wall away from the heart during radiation therapy, resulting in a reduction of the cardiac dose [68]. Nevertheless, the most technologically advanced methods nowadays rely on image-guided and stereotactic body radiation therapies, which enable physicians to precisely deliver radiation beams with higher doses to small, localized areas in tissues [69].

Above all, at present, the most cutting-edge methodology in radiation therapy is FLASH radiotherapy. Although it is still in its preclinical stages, this emerging modality in radiation therapy holds promise for sparing cardiac tissue in thoracic patients treated with radiation. The most notable advantage of FLASH therapy compared to older modalities is its ability to deliver ultra-high doses of radiation in an extremely short span of milliseconds. This mode of radiation specifically targets the tumor tissue with very high-intensity beams and, at the same time, is protective of neighboring non-tumor tissues and organs, sparing them from the toxic effects of radiation [70]. For the time being, FLASH therapy is still not accessible in clinical settings despite the fact that it has been proven to be both feasible and safe, with a more favorable outcome in comparison to other radiation modalities. Many studies conducted during the last decade have suggested that FLASH radiation, which involves delivering doses of radiation at a rate of 40 Gy/s, significantly reduces normal tissue damage while enhancing the therapeutic efficiency of radiotherapy as well as the tumor’s response to this particular line of treatment.

However, to date, only one study demonstrates the cardioprotective benefits of FLASH therapy compared to conventional methods of radiotherapy. This could be considered an important drawback when assessing the reliability of the latter technique; nevertheless, a significant amount of consistent data is being generated nowadays through multiple ongoing clinical trials that are conducted based on this same topic of interest [71]. Notably, recent research employing FLASH proton radiotherapy has shown that this procedure has the potential to preserve major cardiac functions in a preclinical mouse model of RIHD [72]. The findings demonstrate that FLASH radiotherapy more effectively preserves cardiac function, as evidenced by echocardiographic analysis, and also reduces the onset of long-term fibrosis [72].

The main body of evidence regarding FLASH therapy was produced by relying on previous data from pre-clinical models that focused on the lung, brain, skin, intestine, and blood tissues [71]. Multiple clinical trials (phase I NCT04986696, NCT05524064; phase II NCT05724875) are underway in order to better describe both the efficiency and toxicity of high dose rates of FLASH therapy compared to conventional radiotherapy methods [71]. The subjects recruited in those studies include cancer patients with bone metastases in the thorax; remarkably, this latter group of patients could be considered as the perfect cohort with whom to study FLASH radiation-induced heart dysfunction in this particular setting.

Despite improvements in radiotherapy techniques that have reduced the incidence of RIHD, complete avoidance of heart radiation exposure remains a challenge. Traditional and innovative therapeutic strategies are being actively investigated to prevent or mitigate RICAD. Statins have the most preclinical data supporting their radioprotective role. Statin therapy has been proven to provide numerous benefits in terms of reducing the endothelial damage seen in RICAD [73].

Over the past decade, several preclinical and clinical studies have indicated that statins may reduce the effects of radiation-induced cell injuries [74]. A recent retrospective cohort study by Boulet et al. [75] focused on the effects of statins on vascular complications in a group of 5718 cancer patients who underwent radiotherapy targeting the thorax, head, and neck. The findings revealed that patients using statins experienced a significantly lower incidence of cardiovascular events. This suggests that statins could play a crucial role in the treatment or prevention of RICAD [75]. Moreover, recent evidence has linked statin therapy to a decreased risk of major adverse cardiovascular events (MACE) among breast cancer patients who underwent breast-conserving surgery followed by adjuvant whole breast radiotherapy. Among the various statins, hydrophilic options like rosuvastatin and pravastatin have demonstrated the most significant cardioprotective effects. These findings highlight the potential of statins and underscore the need for further research to optimize statin therapy for breast cancer patients receiving radiotherapy due to the absence of any clinical data at this level [76]. Similar to statin therapy, there is a limited amount of clinical data regarding the efficacy of aspirin in modulating and preventing RICAD [74]. Nevertheless, preclinical studies have shown that aspirin can provide radioprotective effects by reducing oxidative damage in rats exposed to lung radiation, suggesting its potential utility as a radioprotector in clinical settings [77]. In contrast, the research conducted by Hoving et al. indicated that while aspirin was effective in alleviating age-related CAD in non-irradiated apolipoprotein E null rats, it did not prevent atherosclerosis in those who underwent radiation therapy [78].

Emerging evidence also suggests that colchicine may be effective as a prophylactic treatment for RICAD [79]. However, the efficacy of these agents has yet to be assessed in prospective human trials, and there are currently no established guidelines for the prophylactic use of medications aimed at preventing the onset of RICAD [74].

Finally, metformin has been reported to reduce the risk of radiation-induced cardiac toxicity in patients with breast cancer [80]. This large-scale cohort study, with a relatively extended follow-up period, assessed the long-term risk of radiation-induced cardiovascular toxicity in patients with breast cancer. Notably, this study demonstrates that metformin used during adjuvant breast radiation therapy may reduce the risk of CAD and HF, particularly in patients with early-stage breast cancer [80]. Therefore, incorporating metformin during adjuvant breast radiotherapy could be considered in clinical practice, as well as in the selection of thoracic radiotherapy protocols that involve scatter irradiation to the heart or coronary arteries (Table 1).

While dose minimization is the most crucial preventive strategy for RICAD, effective prevention starts before treatment initiation and continues through regular clinical evaluations and proactive risk factor modifications. Conventional risk factors, in fact, significantly increase the likelihood of developing RICAD; thus, managing these factors holds substantial promise for lowering RICAD risk. The presence of at least one classic risk factor during irradiation has been associated with a 1.6-fold increase in CAD events among Hodgkin lymphoma survivors, with hypertension nearly doubling this risk [25]. In an earlier study involving patients with the same type of cancer, the presence of one or more risk factors at an 11-year follow-up was linked to a more-than-2-fold increase in CAD incidence [81].

A population-based retrospective study found a 2-fold increase in major coronary events in breast cancer patients, with at least one conventional risk factor present at the start of radiotherapy, although this risk did not appear to be exacerbated by radiotherapy itself [82]. Additionally, a sedentary lifestyle should be recognized as a risk factor for CAD in irradiated patients; adherence to national exercise guidelines correlated with relative risk reductions of 25% and 50% for Hodgkin’s and breast cancer survivors, respectively [83]. Radiotherapy has also been shown to elevate the relative risk of CAD by up to 60% in patients with pre-existing ischemic heart disease [84].

**Figure 2:**
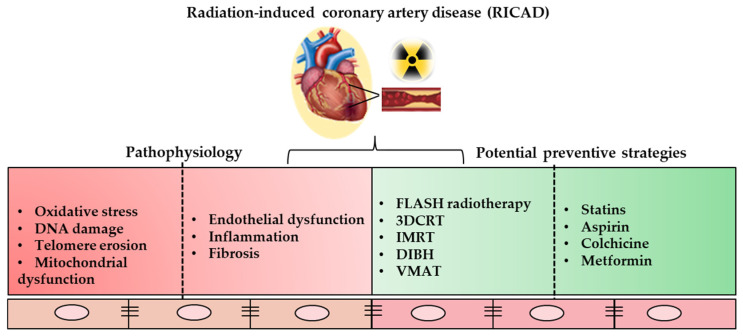
Schematic representation of the biological mechanisms (in red) underlying RICAD, as well as potential preventive strategies (in green). The biological mechanisms include the molecular and cellular processes involved. The preventive strategies encompass radiotherapy techniques and pharmacological interventions aimed at reducing RICAD. 3DCRT: three-dimensional conformal radiation therapy; IMRT: intensity-modulated radiation therapy; DIHB: deep inspiration breath hold; VMAT: volumetric modulated arc therapy.

Currently, there are no established guidelines regarding optimal blood pressure, diabetes control, or the use of statins and anti-hypertensives in post-radiotherapy patients. Furthermore, cardiovascular risk calculators like QRISK2 and SCORE2 do not account for patients’ cancer treatment histories, potentially leading to an underestimation of their cardiovascular risk [3,85]. Thus, it could be more reliable nowadays to integrate additional cancer-related patient details as crucial parameters for the development of much more accurate risk calculators that are better tailored to predict the overall risk of CVD in different patients based on their various backgrounds. While a few studies have developed risk scores specifically for oncology patients, none have yet been prospectively validated [3]. Therefore, assessing cardiovascular risk in post-radiotherapy patients necessitates a tailored approach, supported by a suggested algorithm for risk assessment. Figure 2 illustrates the cellular and molecular mechanisms underlying RICAD, as well as potential strategies for its prevention.

## 5. Conclusions

Until today, radiotherapy is still considered one of the most accessible and efficient therapeutic methods for the treatment of cancer patients. However, despite being both effective and successful in prolonging the lifespan of patients, it unfortunately comes with multiple side effects, which mainly include an increased risk for the development of cardiovascular complications and RICAD. Particularly, it has been shown that chest cancer patients exhibit an increased risk of heart disease. Thus, it is becoming evident that the risk of CAD is increased in cancer survivors and that there is a linear relationship linking cancer survivorship to CAD burden. Although some advances in radiotherapy technologies have been optimized to reduce normal tissue exposure to radiation, this topic is still considered highly challenging, especially since recent epidemiological studies have shown an increased risk of heart disease, even in subjects who are exposed to low doses of ionizing radiation. Therefore, newer strategies are urgently needed to control atherogenesis and CAD in cancer survivors. Additionally, what can be more challenging in this particular context is the diagnosis of CAD in this cohort of patients due to the silent nature of the disease, making it difficult to detect early. Hence, proper screening strategies may be highly valuable in diagnosing RICAD at earlier stages, potentially leading to a reduction in the mortality and morbidity that surround the complications of radiotherapy. On the other hand, despite the fact that many studies have been conducted to better understand and elucidate the pathophysiological processes behind the development of RICAD, a significant gap in our knowledge still exists regarding the relationship between radiotherapy and its cardiovascular effects and complications. In this paper, we have selectively tackled several common mechanisms that may be linked to the pathological progression of RICAD. We also discussed the role of various therapeutic agents, as well as newer radiotherapy methods, in mitigating the negative effects of radiation-induced cardiovascular damage with the aim to develop better treatment procedures for this disease. On this particular note, randomized controlled trials are highly required at this stage in order to confirm any radioprotective effects that may be due to the intervention method or treatment regimen being tested. Thus, our study has some limitations. First, as already mentioned, there is a significant lack of clinical trials pertaining to this particular subject. Also, the trials that should be conducted at this level must consider many parameters in order to avoid misleading conclusions. Hence, any potential results should be carefully analyzed and extrapolated depending on the original ethnicity of the population where the study is conducted. Furthermore, comorbidities should also be cautiously diagnosed and analyzed in this specific context, where patients are randomly selected and interviewed according to the practices of the Bureau of National Health Institute in order to ensure the correctness of the whole procedure. For instance, many cancer registry databases lack important information concerning numerous risk factors that are associated with CVD, including lifestyle and dietary habits, body mass index, and socioeconomic status, which may affect the conclusions of any conducted trial. Accordingly, the process of patient selection is essential when leading such large-scale randomized clinical trials to maintain the reliability of the investigation.

Overall, more research is required to better clarify the etiology of RICAD, as well as its potential treatment strategies; indeed, CAD induced by radiation is a multifaceted phenomenon that requires the unified efforts of both cardiologists and oncologists, as well as those of basic and clinical researchers, in order to come up with the right formula with which to win this race.

## Figures and Tables

**Table 1:** A list of potential preventive strategies for the treatment of radiation-induced coronary artery disease and their different modes of action.

Preventive Strategy	Mode of Action
Three-dimensional conformal radiation therapy (3DCRT)	Relies on computed tomography technology for more accurate delivery
Intensity-modulated radiation therapy (IMRT)	Simultaneously delivers multiple radiation beams with variable individual intensities; is very efficient; spares non-target tissues
Deep-inspiratory breath hold (DIBH)	Displaces the breast and chest wall away from the heart during radiation therapy; reduces cardiac dose
FLASH radiotherapy	Delivers ultra-high doses of radiation in an extremely short span of milliseconds; specifically targets the tumor tissue; protective for neighboring non-tumor tissues
Statins	May reduce the effects of radiation-induced cell injuries
Aspirin	Can provide radioprotective effects by reducing oxidative damage
Metformin	May reduce the risk of radiation-induced cardiac toxicity in patients with breast cancer

## Abbreviations

The following abbreviations are used in this manuscript:

3DCRT	Three-dimensional conformal radiation therapy
CAD	Coronary artery disease
CVD	Cardiovascular disease
DAMPs	Danger-associated molecular patterns
DIBH	Deep-inspiratory breath hold
DSBs	Double-stranded breaks
HF	Heart failure
HUVEC	Human umbilical vein endothelial cell
ICAM-1	Intercellular adhesion molecule-1
IMRT	Intensity-modulated radiation therapy
LET	Linear energy transfer
MACE	Major adverse cardiovascular events
mtDNA	Mitochondrial DNA
NF-κB	Nuclear factor-kappa beta
PAI-1	Plasminogen activator inhibitor-1
RICAD	Radiation-induced coronary artery disease
RIHD	Radiation-induced heart disease
ROS	Reactive oxygen species
SASP	Senescence-associated secretory phenotype
SSBs	Single-stranded breaks
VCAM-1	Vascular cell adhesion molecule-1
VHD	Valvular heart disease
VMAT	Volumetric modulated arc therapy
